# False Impressions

**Published:** 1889-02-02

**Authors:** Caroline Fothergill

**Affiliations:** Author of "An Enthusiast," "A Voice in the Wilderness," etc.


*(To be continued.)*

### FALSE IMPRESSIONS.

By Caroline Fothergill, Author of "An Enthusiast," "A Voice in the Wilderness," etc.

*(Continued from p. 272.)*

### Chapter XVII.—Scheming.

Beatrice's first impulse was to refuse Mrs. Lester's invitation. Somehow it offended her pride, and there was something about the whole affair which puzzled, and in a vague, only half-understood way, alarmed her. All her acquaintance and intercourse with George had been so strange, so different from all her previous experiences. Their meeting and recognition of one another before they became formally acquainted, then the antagonism which had lurked under the surface polish of their manner, George's avoidance of her, and now this sudden rush for her acquaintance. She did not understand it; she did not know what was his object in behaving as he had done, and she did not feel inclined to maintain a passive attitude, while he behaved just as he chose. She felt as if she were being drawn into a position which she might afterwards find disagreeable, but from which she could not withdraw. As long as George had stood aloof, she had wished to advance; as soon as he approached, she wished to retreat, or at any rate to stand still. Her first idea, then, was to refuse this invitation, and she said so decidedly. Laura implored her to accept it; she said Mayfield was so stagnant in July, it would be "flying in the face of Providence" to refuse so promising an opportunity for an evening's entertainment. Laura was, as beforesaid, deeply interested in the whole business, she was also extremely amused with both Edith and Mrs. Lester, and amusement was one of the chief aims of her life. She opposed Beatrice's objections with great skill, and at last came off triumphant.

"Why should we not go?" she asked.

"It is not just the going," answered Beatrice; "it is what it will lead to: this will be only the beginning; we shall have to ask them back, and then, I suppose, we shall go on asking one another."

"Well, why should we not?" asked Mrs. Ledward again, with an air of the utmost innocence and simplicity; "why should we not know them? There is nothing wrong about them, and they have at least the merit of being one and all highly amusing."

Beatrice said nothing, but she was not convinced. She could not give her own private reasons, which were nevertheless all-sufficient to her, so she was forced to be silent, and Laura, by ignoring the fact that she could have any private reasons, triumphed.

Mrs. Ledward and George were the only members of these two households who really enjoyed this dinner party. Mrs. Lester had said as much more as she dared, to bring her brother to a better frame of mind as regarded Mrs. Hunter and Mrs. Williamson, but in vain. Edith was made unhappy by her aunt's ill temper and constant directions as to what she might do and what she might not. Beatrice was wishing all the time that she had not yielded to Laura.

She had not given a thought to the probable arrangement of the guests, and, seeing one or two ponderous and handsomely-dressed dowagers present, experienced a degree of surprise when, on the announcement of dinner, George offered her his arm. She rose slowly from her seat, and rested no more than the tips of her delicately-gloved fingers on his arm. It was a large party, and the long string of guests filed in at the tail of her sweeping gown of grey brocade. Mrs. Ledward felt a thrill of admiration as she contrasted Beatrice's queenly figure with the matronly and, in many cases, shapeless forms of the other ladies present; and Edith, with her hand already on Harry Fairfax's arm, suddenly realised what a striking sight was that of Miss Stanford and Mr. Murray standing together.

Beatrice felt the reverse of socially inclined, and her first replies were given in the curt tones she often used; but, however much she might deny it, both to herself and Laura, George's influence over her was very strong. He was happy and in good spirits, and almost insensibly her manner thawed, until she became all at once the charming woman she naturally was. George spoke to no one else, they talked to one another, and seemed scarcely to know that there was anyone else present. Mrs. Lester saw it, and grew more and more annoyed. She could scarcely sit still in her chair, or fulfil her duties, or pay any heed to what was said to her. Her one desire—and she could have wept with vexation that she might not yield to it—was to sit and watch these two who were so absorbed in one another. Others noticed them too: she saw people glancing at them; she knew her two old friends, Mesdames Hunter and Williamson, who were acquainted with her anxiety on her brother's behalf, would not fail to take the earliest opportunity of revenging themselves for being allotted lower places at the feast by condoling with her in the change which was imminent. She could not deny, either, how well they looked together. Beatrice had seldom looked more beautiful, even in Laura's prejudiced eyes, and George, interested and animated, was looking as nearly handsome as he ever did.

It is a pity Edith saw so little of this; it might have opened her eyes, and saved her from the mistake she afterwards made. She was looking exquisitely pretty this evening; George had paid her a very happy compliment on her dress and appearance, and Harry Fairfax was delighted at the idea of having her by his side so long. It may be said here that the curate was a very good-looking young man, though with rather a boyish face and manner; he was very popular with the ladies of his parish, and, as we know, Edith had a very kindly feeling for him; but now it was becoming overshadowed by her growing attachment to George, and this evening she found him decidedly wanting. On so solemn an occasion as her first dinner party she thought he lacked dignity, and was altogether too lively. Then, too, he would talk in a way which made it necessary that she should answer, and give him almost all her attention. She wished he was a little more like Mr. Murray—if only he would not speak so enthusiastically and move so unnecessarily in his chair, and talk so incessantly. She sighed and began to answer a little at random, until he pulled her up abruptly and showed her he meant to have all her attention. She was dimly aware that he was "paying her attentions," and up to a very recent date those attentions had been very agreeable to her, but now she had lost all taste for them, and only wondered how she could ever have felt flattered and pleased by them. Just now her thoughts ran on deep dark eyes, and an interesting wanness of complexion, coupled with a fascinating smile and a certain gentle masterfulness of manner, which, while seeming to persuade, should yet carry all before it in a perfectly irresistible way. Mr. Fairfax fulfilled none of these requirements. He was not particularly tall, he was fair and of a ruddy countenance, with honest grey eyes, more suggestive of fun than "depth." His manners were boyish and cheerful, so as to be absolutely commonplace; his nature was more kind than masterful. He was very fond of tennis, and would play until his face was crimson, and he had to mop it with a handkerchief. Edith could not make a hero of him, and she found the time spent at the dinner-table rather long.

The party broke up soon after ten. George said "Good-night" to his sister and Edith as soon as the door had shut behind the last guest, and went away to his study to finish some writing. Edith, too, was tired, and as soon as she had done one or two little things in the drawing-room, which Mrs. Lister disliked leaving to the servants and had given into her niece's care, she went to her room, and Mrs. Lester was left alone.

She was not tired, on the contrary she felt almost preternaturally wide awake. Nevertheless, she went to her bedroom and summoned her maid as usual. After she had sent her away again, she sat in her wrapper in an easy-chair and began to think out her position. Her brother was in love; if she had suspected it before, she was sure of it now, and the next step would be his marriage. Whether Miss Stanford returned the feeling she did not stay to inquire—she would have said there was no need. Her brother's marriage would mean her expulsion from Queen Street, therefore the marriage must be hindered. She thought and thought with the intensity and concentration of despair. Her own home was in danger; she would have to make another for herself, and although she shrank from acknowledging it even to herself, deep down in her consciousness was the certainty that when once she had left her brother's house her life would be exceedingly lonely; that she had not one acquaintance who sought her society for her own sake. It was a bitterly humiliating thing to have to confess even to herself. The thought of her barren and isolated life was intolerable; she could not face it, and she set her lips stubbornly, and bent all her mind to the resolution of the problem how it was to be prevented. For a long time she thought in vain. To offer any objection to her brother, even if she could summon up sufficient courage, would be to simply defeat her own purpose, and to hurry on the catastrophe. She could not speak to Beatrice. Although she would have angrily repudiated any such suggestion, she stood in some awe of Miss Stanford, feeling in her presence the discomfort and uneasiness which an habitually crooked nature feels in the presence of a straightforward one. The idea of Beatrice stepping into the position she had occupied for so long was inexpressibly galling to her. Let him marry anyone else, if he must marry, only not her.

She thought and thought—one plan after another was rejected; she felt desperate. Long after midnight she still sat there, lost in thought, a strange and unsightly object, in the luxuriously furnished room. Her scanty locks were arranged for the night, her thin figure and yellow face were in sharp contrast to the white wrapper she wore, her brows were contracted, her dull grey eyes fixed with the intensity of her thought. She looked what she was—a hard, unloved, selfish woman.

Suddenly her brows relaxed, her face cleared, her lips parted in a half smile; she drew herself erect with a sigh of relief, saying, half aloud:

"That will do; it is just what I want. I wonder I did not think of it before."

And as the plan was a thoroughly wicked and heartless one, perhaps it is wonderful she did not think of it before.

She would try to turn George's thoughts from Beatrice by giving him someone else to think about, and that someone was close by, "made to her hand." It was Edith. She had noticed Edith's attachment to George, and had more than once thought of warning her not to let her feelings become too strong for her. She was glad now she had not done so. She knew her brother had a very kindly feeling for Edith, which was quite enough, considering Edith's state of mind, to give an air of probability to what she said. She would give Edith to suppose—a few hints would be enough, she need tell no absolute untruths—that George was seriously attached to her; that would put the crowning point to Edith's infatuation. She would throw them together and fan the flame as much as she could, she would be prepared to betray Edith's feelings to George, to deliberately compromise the girl if she could compass her end in no other way. He might marry Edith and welcome, she would never leave the house on her account. She believed Edith herself would ask her to stay.

This was her plan to secure her own comfort, and save her vanity. It is difficult to say how she justified her conduct even to herself. She was punctilious in the performance of her religious duties. She read a portion of Scripture morning and evening, she went to church regularly twice every Sunday, she subscribed to various missionary societies, also to the societies for the prevention of cruelty to animals and children. From a moral point of view, generally, she had a very high opinion of herself. But self was paramount, and having, as far as she could, thought out the details of her scheme, she sought her couch with a clear conscience.

